# Supplementary material for: Comparative multiomics analysis of cell physiological state after culture in a basket bioreactor
Source: Sci Rep. 2022 Nov 23;12:20161. doi: 10.1038/s41598-022-24687-4 (PMC9686226; doi:10.1038/s41598-022-24687-4)
Supplement: Supplementary file 1 — Supplementary Information 1. [file 41598_2022_24687_MOESM1_ESM.zip › raw data/Metabolomics raw data/5.MetKeggEnrichment/EG.vs.CG/EG.vs.CG_pos.KEGG_Enrich.scatterplot.pdf]

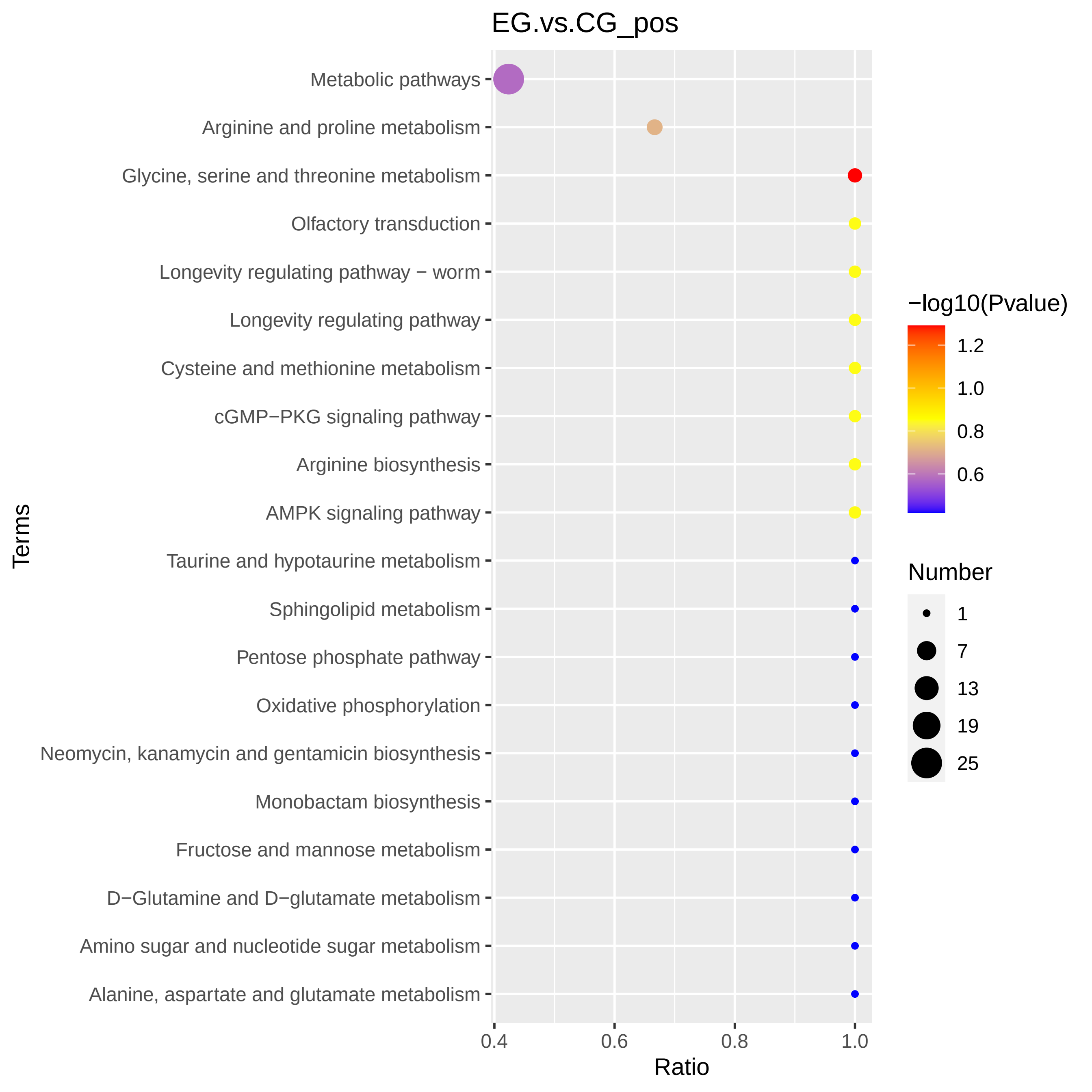

EG.vs.CG_pos
Metabolic pathways
Arginine and proline metabolism
Glycine, serine and threonine metabolism
Olfactory transduction
Longevity regulating pathway − worm
Longevity regulating pathway
Cysteine and methionine metabolism
cGMP−PKG signaling pathway
Arginine biosynthesis
AMPK signaling pathway
Taurine and hypotaurine metabolism
Sphingolipid metabolism
Pentose phosphate pathway
Oxidative phosphorylation
Neomycin, kanamycin and gentamicin biosynthesis
Monobactam biosynthesis
Fructose and mannose metabolism
D−Glutamine and D−glutamate metabolism
Amino sugar and nucleotide sugar metabolism
Alanine, aspartate and glutamate metabolism
Terms
0.4
0.6
0.8
1.0
Ratio
−log10(Pvalue)
1.2
1.0
0.8
0.6
Number
1
7
13
19
25
